# Biomimetic Design and Fabrication of Sericin-Hydroxyapatite Based Membranes With Osteogenic Activity for Periodontal Tissue Regeneration

**DOI:** 10.3389/fbioe.2022.899293

**Published:** 2022-05-19

**Authors:** Piaoye Ming, Pengcheng Rao, Tianli Wu, Jianghua Yang, Shi Lu, Binbin Yang, Jingang Xiao, Gang Tao

**Affiliations:** ^1^ Luzhou Key Laboratory of Oral and Maxillofacial Reconstruction and Regeneration, The Affiliated Stomatological Hospital of Southwest Medical University, Luzhou, China; ^2^ Department of Oral Implantology, The Affiliated Stomatological Hospital of Southwest Medical University, Luzhou, China; ^3^ Department of Oral and Maxillofacial Surgery, The Affiliated Hospital of Southwest Medical University, Luzhou, China

**Keywords:** sericin, nano-hydroxyapatite, biomimetic membranes, human periodontal membrane stem cells, osteogenic differentiation

## Abstract

The guided tissue regeneration (GTR) technique is a promising treatment for periodontal tissue defects. GTR membranes build a mechanical barrier to control the ingrowth of the gingival epithelium and provide appropriate space for the regeneration of periodontal tissues, particularly alveolar bone. However, the existing GTR membranes only serve as barriers and lack the biological activity to induce alveolar bone regeneration. In this study, sericin-hydroxyapatite (Ser-HAP) composite nanomaterials were fabricated using a biomimetic mineralization method with sericin as an organic template. The mineralized Ser-HAP showed excellent biocompatibility and promoted the osteogenic differentiation of human periodontal membrane stem cells (hPDLSCs). Ser-HAP was combined with PVA using the freeze/thaw method to form PVA/Ser-HAP membranes. Further studies confirmed that PVA/Ser-HAP membranes do not affect the viability of hPDLSCs. Moreover, alkaline phosphatase (ALP) staining, alizarin red staining (ARS), and RT-qPCR detection revealed that PVA/Ser-HAP membranes induce the osteogenic differentiation of hPDLSCs by activating the expression of osteoblast-related genes, including ALP, Runx2, OCN, and OPN. The unique GTR membrane based on Ser-HAP induces the differentiation of hPDLSCs into osteoblasts without additional inducers, demonstrating the excellent potential for periodontal regeneration therapy.

## Introduction

The repair of periodontal bone defects caused by periodontitis, infection, and trauma remains a challenge in clinical treatment (Orlandi et al., 2021). The guided tissue regeneration (GTR) technique is the mainstay therapy for periodontal bone defects. This generally combines bone meal with a GTR membrane to promote alveolar bone reconstruction. GTR membranes are most frequently used to restrain the ingrowth of fibrous tissue and create a suitable space for alveolar bone regeneration (Aral et al., 2020; Zhang et al., 2021; Zheng et al., 2021). Ideal GTR membranes should have an appropriate mechanical strength and be able to induce the osteogenic differentiation of autologous stem cells at the site of the alveolar bone defect (Liao et al., 2021; Woo et al., 2021). Currently, GTR membranes are prepared by combining the advantages of different biomaterials into composites. Inorganic materials (HAP, Zinc, tricalcium phosphate, etc.) and polymers (chitosan, gelatin, PCL, PLGA, etc.) were used for periodontal tissue regeneration. Wu et al. successfully fabricated biocompatible chitin hydrogel films incorporated with ZnO and found that composite GTR membrane has excellent antibacterial abilities and can effectively promote periodontal repair (Wu et al., 2021). However, the most frequently used GTR membranes still have limitations, such as high cost, poor mechanical properties, and a lack of osteogenic activity. Therefore, the development of low-cost GTR membranes with good biocompatibility and excellent osteogenic function has attracted increasing attention (Nguyen et al., 2021; Tedeschi et al., 2021).

Sericin, a soluble protein extracted from domestic silkworm cocoons, contains 17–18 amino acids, including glycine, serine, alanine, and tyrosine. Due to its high carboxyl, amino, and hydroxyl group content, sericin has exhibited diverse biological activities, including anti-apoptosis and anti-oxidation properties (Zhao et al., 2014; Lamboni et al., 2015; Li et al., 2021). In addition, the biological activity of sericin in promoting cell migration and differentiation has been demonstrated (Martínez-Mora et al., 2012; Nayak et al., 2013). Sericin has excellent biocompatibility and biodegradability, making it attractive for the development of tissue engineering materials. The application of sericin has been studied for a range of tissue repairs, including sericin catheters for peripheral nerve regeneration, sericin scaffolds for cartilage repair, and sericin hydrogels for wound repair. Qi et al. (2020) prepared a photo-crosslinked sericin/graphene oxide hydrogel and found that the sericin composite scaffold exhibited good biocompatibility, cell adhesion, proliferation, and osteogenic induction property. However, the use of sericin as a GTR membrane for periodontal bone repair has not yet been explored. Therefore, we aimed to use sericin to prepare a GTR membrane with osteogenic properties, with the objective of effectively promoting the osteogenic differentiation of endogenous stem cells and the repair of periodontal bone defects.

Hydroxyapatite (HAP) is the most commonly used osteogenic material in bone tissue engineering owing to its similar biodegradability, crystal structure, and chemical properties to those of biological apatite (Fihri et al., 2017). HAP simultaneously releases Ca and P during its degradation process, giving it the potential to promote bone regeneration in bone tissue engineering (D'Elia et al., 2020). The formation of bone involves a series of complex events, with the critical step being the mineralization of calcium phosphate on the extracellular matrix (ECM) to form HAP crystals. Numerous natural and synthetic polymers have been used as templates for growing HAP and creating scaffold materials with osteogenic activity. Recently, hydroxyapatite fabricated using proteins has attracted widespread attention as an organic template (Raina et al., 2020; Bal et al., 2021; Zhu et al., 2021). Cai et al. (2010) showed that the crystallization of HAP could be affected by introducing proteins into the system. Tao reported a simple, one-step, “green” synthetic strategy using sericin as a biomineralized template for AgNPs synthesis to prepare sericin-AgNPS sponge dressings. The dressing has excellent biocompatibility and antimicrobial activity properties making it a competitive candidate for an effective wound dressing (Tao et al., 2019). Proteins contain a variety of active groups that can be used as templates for HAP deposition in the formation of protein/HAP nanocomposites. Similarly, sericin protein can be used as a biomineralization template for the deposition of HAP.

**SCHEME 1:**
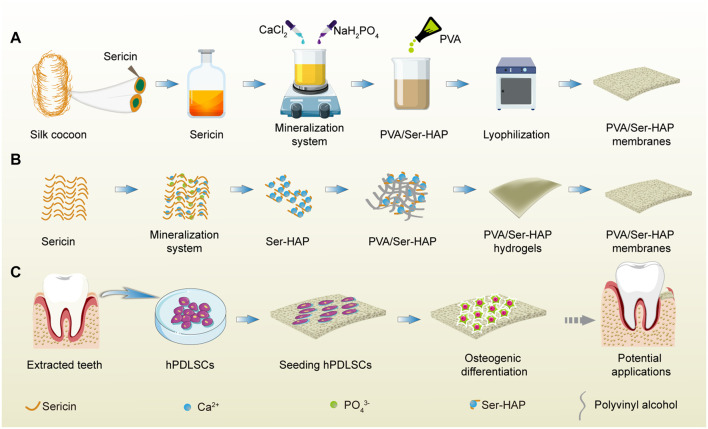
A schematic illustration of the fabrication and application of PVA/Ser-HAP membrane. **(A)** The preparation process of PVA/Ser-HAP membrane; **(B)** Proposed schematic diagram depicting sericin-mediated nucleation of HAPs and preparation of PVA/Ser-HAP membrane; **(C)** The osteogenic differentiation of hPDLSCs on mineralized PVA/Ser-HAP membrane and potential periodontal tissue regeneration application for PVA/Ser-HAP membrane.

In this study, the effect of sericin on the mineralization of HAP was investigated, and it was found that increasing the sericin concentration led to a gradual decrease in HAP particle size. Polyvinyl alcohol (PVA), a biocompatible, biodegradable, non-toxic, and hydrophilic synthetic polymer, was widely used to prepare biomedical materials. It is a semi-crystalline polymer with excellent strength and flexibility that are crucial for biomaterials applications (Pan et al., 2021; Zhou et al., 2021). Polyvinyl alcohol (PVA) was combined with Ser-HAP and subjected to three freeze/thaw cycles to prepare PVA/Ser-HAP membranes (Scheme 1A,B). FESEM was used to characterize the morphological features of the PVA/Ser-HAP membranes. ARS and ALP staining were used to investigate the effect of the PVA/Ser-HAP membranes on the osteogenic differentiation of hPDLSCs. In addition, the expression of osteogenic-related genes, including ALP, runt-related transcription factor (Runx2), osteopontin (OPN), and osteocalcin (OCN) were assayed. The feasibility of using the PVA/Ser-HAP membrane as a new GTR membrane was verified (Scheme 1C). This study provides substantial support for the biomimetic design and preparation of sericin-based GTR membranes.

## Materials and Methods

### Materials

Silkworm cocoons were obtained from the Seri Cultural Research Institute, China Academy of Agriculture Science (Jiangsu, China). Calcium chloride (CaCl_2_), polyvinyl alcohol (PVA), and monosodium phosphate (NaH_2_PO_4_) were purchased from Macklin Biochemical Technology Co., Ltd. (Shanghai, China). Sodium carbonate anhydrous (Na_2_CO_3_) was purchased from Solarbio Technology Co., Ltd. (Beijing, China). And sodium hydroxide (NaOH) was obtained from Kelong Chemicals Co., Ltd. (Chengdu, China). All other reagents, including α-modified eagle’s medium (α-MEM; Gibco, CA, United States), fetal bovine serum (FBS; Gibco, United States), 1% penicillin-streptomycin (Beyotime, United States), 0.25% trypsin-EDTA (Gibco, CA, United States), 4% paraformaldehyde (Sigma-Aldrich), Live/Dead® Viability Kit (Thermo Fisher Scientific, United States), Cell Counting Kit-8 (CCK-8, APE-BIO, United States), BCIP/NBT alkaline phosphatase color development kit (Beyotime, China), alizarin red staining solution (ARS, Cyagen, China), Total RNA extraction kit (TianGen, China), and RevertAid First Strand cDNA Synthesis Kit (Thermo Fisher Scientific, Waltham, United States), were used directly without further purification.

### Isolation of Sericin

Sericin was extracted from silkworm cocoons using a previously described high temperature and alkaline degumming method (Tao et al., 2021). Briefly, 10 g of silkworm cocoon was weighed and washed three times with deionized water. The pre-prepared cocoons were then placed in 250 ml of ultrapure water containing 2% (w/v) sodium carbonate. The mixture was allowed to boil at 120°C for 30 min to remove silk fibroin. The solution was then filtered and centrifuged at 3000 rpm for 5 min to remove insoluble residues and then dialyzed (MWCO: 3500 Da) for 48 h to remove hetero-ions. Finally, the sericin powder was obtained by freeze-drying and stored at 4°C before use.

### Synthesis and Characterization of Sericin-HAP Particles

Sericin protein-mediated HAP mineralization was carried out using the co-precipitation method. Sericin concentrations of 0, 10, 50, and 100 μg/ml were used to investigate the effect of sericin concentration on the mineralization of HAP. Briefly, 2.22 g of CaCl_2_ was dissolved in 50 ml of deionized water with various amounts of sericin to give solution A, and 1.44 g of NaH_2_PO_4_ was dissolved in an equal volume of deionized water (50 ml) to give solution B. The two solutions were then combined, heated to 52°C, and stirred for 2 h. Throughout the process, the pH of the mixed solution was measured using a pH Tester 10 (Eutech Instruments, United States) and adjusted with 0.1 M NaOH to maintain a pH of 11 ± 0.1. The final solution was aged at 4°C for 24 h, and then dried at 60°C for 24 h. The four resulting composites were denoted Ser0-HAP, Ser10-HAP, Ser50-HAP, and Ser100-HAP according to the concentration of sericin used: 0, 10, 50, and 100 μg/ml, respectively. For the Ser100-HAP group, the settling time was extended from 2 to 8 h to explore the effect of mineralization time on HAP nucleation.

The morphology and elemental composition of the Ser-HAP particles were determined using a transmission electron microscope (TEM, JEM-2100, Japan) equipped with energy dispersive spectroscopy (EDS). X-ray photoelectron spectroscopy (XPS, Shimadzu Kratos AXIS Ultra DLD, Nagoya, Japan) was used to assess the surface elemental composition. Fourier transform infrared (FTIR, Thermo Fisher Scientific, MA, United States) spectra in the range 4000–500 cm^−1^ with a resolution of 2 cm^−1^ were used to verify the surface functional groups. X-ray diffraction (XRD, PANalytical X'Pert powder, Almelo, Netherland) in the 2θ range 10°–70° was used to verify the formation of HAP particles.

### Fabrication and Characterization of the PVA/Ser-HAP Membranes

PVA (4%, w/v) solution was prepared by dissolving 4 g of PVA in 100 ml of deionized water and stirring at 100°C for 1 h to allow complete dissolution. The 1% (w/v) sericin solution was mixed with (4%, w/v) PVA solution at a 1:1 ratio at 80°C with continuous stirring for 30 min Ser100-HAP particles were then added to the mixture and stirred for 30 min. The concentration of Ser100-HAP particles was 0, 10, 50, or 100 μg/ml, and the corresponding membranes were denoted PVA/Ser-HAP0, PVA/Ser-HAP1, PVA/Ser-HAP2, PVA/Ser-HAP3, respectively. The mixtures were then poured into a 24-well plate to form PVA/Ser-HAP membranes. The membranes were frozen (−20°C) and thawed 3 times to improve their mechanical properties. Finally, the composite membranes were obtained by freeze-drying for 24 h using a SCIENTZ-12N Freezer Dryer (Ningbo, China). The PVA/Ser-HAP membranes were coated with platinum, and then the surface topography was analyzed by FESEM. The trace elements in the membrane were analyzed using energy dispersive spectroscopy (EDS).

### Hydrophilicity and Degradation of PVA/Ser-HAP Membranes *In Vitro*

Composite membranes with the same dimensions were immersed in deionized water at 37°C. The initial dried weight was denoted W0. The swollen weight was determined when the swelling became stable and designated W1. The swelling ratio (SR) was calculated using Eq. 1.

SR(%)=(W1−W0)/W0×100% (1)

On days 0, 7, 14, 21, 28, 35, and 63, the samples were dried. The weight was determined and designated W2. The degradation ratio (DR) was then calculated using Eq. 2 (Thangprasert et al., 2019; Prakash et al., 2020).

DR(%)=(W0−W2)/W0×100% (2)

### Culture and Identification of hPDLSCs

This study was approved by the ethics committee of Southwest Medical University. Informed consent was obtained from all of the human participants. Healthy teeth were collected from five people receiving orthodontic treatment in the Department of Oral and Maxillofacial Surgery, The Affiliated Stomatological Hospital of Southwest Medical University. The tissue blocks were isolated from the root surface but not the apex, washed and cultured in α-modified eagle’s medium, and incubated in a humidified atmosphere with 5% CO_2_ at 37°C. The medium was changed every 3 days until the cells reached 80% confluency. The hPDLSCs were then purified by monoclonal culture as previously described (Zhu and Liang, 2015; Li et al., 2017; Li et al., 2021). In short, P0 cells were inoculated in 96-well plates at a concentration of 500 cells/mL for single cell-derived colony selection. The cells were then sub-cultured with 0.25% trypsin-EDTA and P3 cells were used in the subsequent experiments.

When the third-generation cells reached 70%–80% confluency, they were treated with 0.25% trypsin-EDTA and washed three times with phosphate buffered saline (PBS). hPDLSCs (5×10^5^/ml) were then incubated with human antibodies for CD31 (FITC), CD34 (APC), CD45 (FITC), CD73 (PE), CD90 (FITC), and CD105 (FITC) (Bio-legend, CA, United States) at 4°C. Flow cytometry (FACSCalibur, CA, United States) was then used to detect stem cell surface markers of the samples. The data were analyzed using Win MDI 2.8 software (The Scripps Institute, West Lafayette, IN, United States).

### Cytocompatibility of Sericin and Ser-HAP Particles

In this study, Live/Dead staining and CCK-8 assay were used to investigate the proliferation of cells treated with sericin and Ser-HAP particles. Briefly, hPDLSCs were inoculated in 24-well plates (5×10^3^ cells/well) and cultured in α-modified eagle’s medium supplemented with 10% FBS and 1% penicillin-streptomycin. The medium was replaced with a serum-free medium containing different concentrations of sericin, while for the Ser-HAP particles, the culture medium was replaced with a complete medium. After co-culturing for 48 h, hPDLSCs were incubated in Live/Dead staining solution for 30 min at room temperature and observed using a fluorescence microscope. A CCK-8 assay was then carried out according to a previously described method (Wang et al., 2015). Briefly, 90 μL of α-modified eagle’s medium was mixed with 10 μL of CCK-8 and incubated for 1.5 h at 37°C. The solutions were then analyzed at 450 nm using a microplate reader (TECAN Infinite M200PRO, China).

### Biocompatibility of the PVA/Ser-HAP Membranes

The PVA/Ser-HAP membranes were sterilized under ultraviolet light for 2 h and placed in 24-well plates. A hPDLSC suspension (0.5 ml, 1×10^5^ cells/mL) was seeded on the membranes. After co-culture for 48 h, the Live/Dead staining of hPDLSCs was performed according to the manufacturer’s instructions. For the CCK-8 assay, the culture fluid was discarded, and α-modified eagle’s medium containing 10% CCK-8 solution was added. After incubation at 37°C for 1.5 h, the solution was added to 96-well plates and measured at 450 nm using a microplate reader (TECAN Infinite M200PRO, China).

### Cell Morphology on PVA/Ser-HAP Membranes

The morphology of hPDLSCs grown on the PVA/Ser-HAP membranes was analyzed using filamentous actin (F-actin) staining. hPDLSCs (2×10^4^ cells/well) were seeded on the PVA/Ser-HAP membranes. After culturing for 3 or 5 days, the membranes were washed three times with PBS and fixed with 4% paraformaldehyde at 4°C for 30 min. The F-actin and cell nuclei of the hPDLSCs were stained with rhodamine phalloidin and 4-6-diamidino-2-phenylindole (DAPI), respectively, for 15 min. The samples were then sealed with 10% glycerin containing fluorescent anti-crack agent. Finally, the morphology of the hPDLSCs was observed using confocal laser microscopy (Nikon, Tokyo, Japan). All the operations were performed in the dark.

### Alkaline Phosphatase Staining

hPDLSCs (2×10^4^ cells/well) were seeded in a 24-well plate and cultured in a non-osteogenic induction medium containing different kinds of Ser-HAP particles. All of the Ser-HAP particles were at a concentration of 50 μg/ml. After co-culture for 3 or 5 days, 4% cold paraformaldehyde was used to fix the hPDLSCs for 15 min. The hPDLSCs were then stained with BCIP/NBT alkaline phosphatase color development kit for 1 h at 37°C.

To evaluate the osteogenic performance of the PVA/Ser-HAP membrane, the non-osteogenic induction medium was first prepared with the extract of the PVA/Ser-HAP membrane. The extract of materials was centrifuged at 3000 r/min for 10 min and filtered by sterilizing filter (Millipore, Canada). Subsequently, hPDLSCs (2×10^4^ cells/well) were seeded in a 24-well plate with normal media and allowed to reach 80% fusion. The normal media were then replaced with the leaching liquors as described above. After co-culture for 3 or 5 days, alkaline phosphatase staining was performed in the same way as described above. For quantification, the data was processed by utilizing ImageJ.

### Alizarin Red Staining

hPDLSCs (2×10^4^ cells/well) were seeded in a 24-well plate and co-cultured with the leaching liquors of the PVA/Ser-HAP membranes as described above. After culturing for 21 days, the hPDLSCs were fixed with 4% cold paraformaldehyde for 30 min at 4°C and then incubated with alizarin red stain at 37°C for 1 h. Subsequently, the calcium deposition was imaged using fluorescence microscopy. For quantification, the data was processed by utilizing ImageJ.

### Osteogenic Gene Expression

Real-time fluorescence quantitative PCR (RT-qPCR) was used to evaluate the expression of the osteogenic genes of hPDLSCs, including ALP, Runx 2, OPN, and OCN. β-Actin was used as a reference gene. Following co-culture for 3 or 5 days, hPDLSCs were lysed with a Total RNA extraction kit and RNA was collected according to the manufacturer’s instructions. The cDNA was then obtained using a Revert Aid First Strand cDNA Synthesis Kit according to the manufacturer’s instructions. All primers were compounded by Shenggong Bioengineering Co., Ltd. (Shanghai, China). The primer sequences for RT-qPCR are shown in Table 1. All experiments were performed in triplicate and analyzed using the 2^−ΔΔCT^ method (Xing et al., 2021).

**TABLE 1:** Primer sequences.

Primer name	Forward (5–3′)	Reverse (5–3′)
Runx2	CAC​TGG​CGC​TGC​AAC​AAG​A	CAT​TCC​GGA​GCT​CAG​CAG​AAT​AA
ALP	GGA​CCA​TTC​CCA​CGT​CTT​CAC	CCT​TGT​AGC​CAG​GCC​CAT​TG
OCN	CTAAGAAGTTTCGCAGAC	TGTCCCAATCAGAAGG
OPN	GAT​GAA​TCT​GAT​GAA​CTG​GTC​ACT​G	GGT​GAT​GTC​CTC​GTC​TGT​AGC​A
β-actin	GAT​GAG​ATT​GGC​ATG​GCT​TT	CAC​CTT​CAC​CGT​TCC​AGT​TT

### Statistical Analysis

All experiments were repeated in triplicate independently. The representative data were manifested by mean ± standard deviation (SD). One-way analysis of variance (ANOVA) was used to analyze the differences between groups. A *p*-value of <0.05 was accepted as statistical significance. (*p < 0.05, **p < 0.01, ***p < 0.001, ****p < 0.001).

## Results and Discussion

### Characterization of Ser-HAP Particles

**FIGURE 1:**
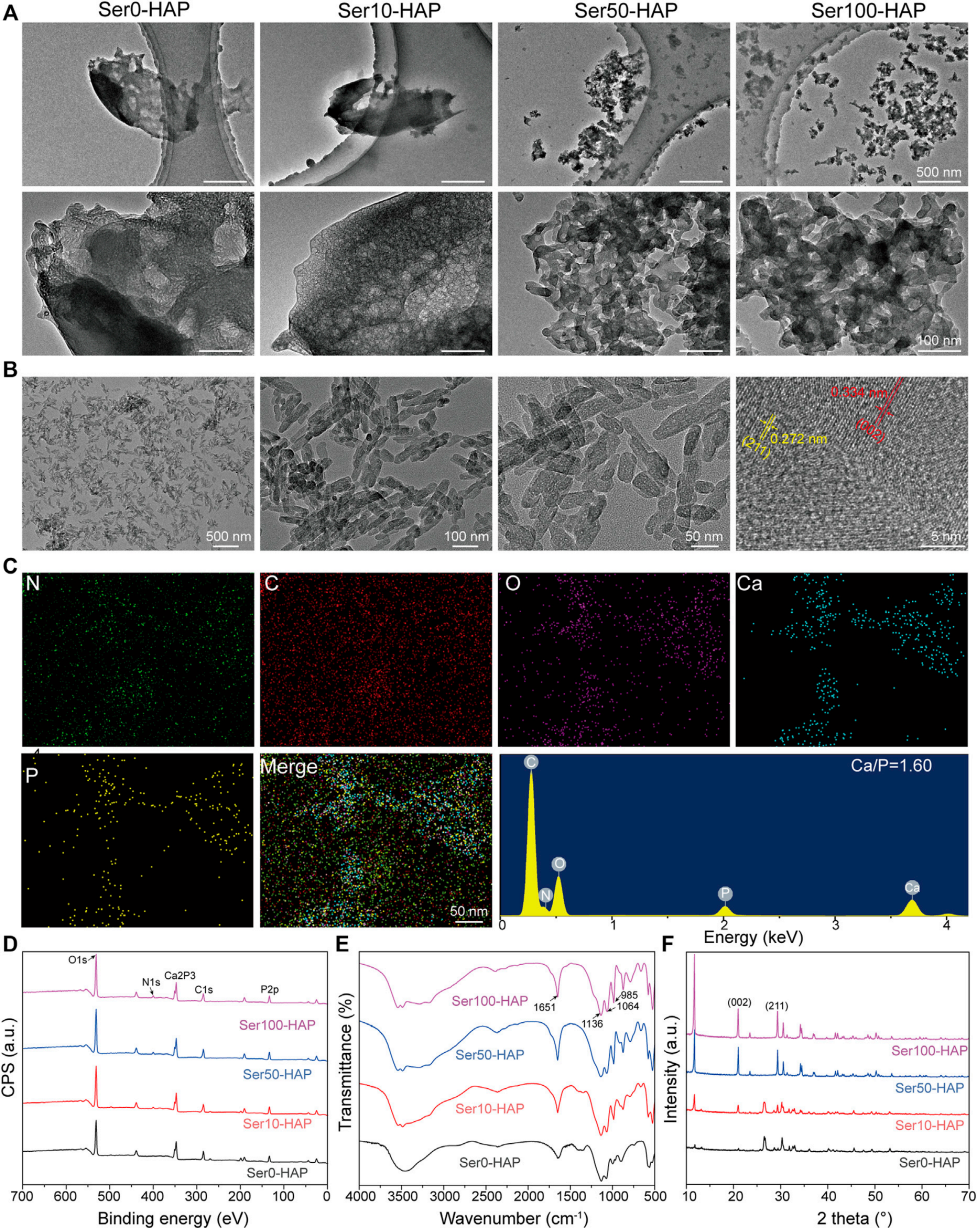
Surface morphology and characterization of Ser-HAP. **(A)** TEM micrographs of Ser-HAP particles; **(B)** TEM and HRTEM images of Ser100-HAP particles; **(C)** EDS analysis of Ser100-HAP particles; **(D)** XPS spectra; **(E)** FTIR spectra; and **(F)** XRD patterns of Ser-HAP particles.

The morphology of the Ser-HAP particles was determined using TEM. As shown in Figure 1A, the Ser-HAP particles gradually decreased in size with increasing sericin concentration from 0 to 100 μg/ml. The 0 and 10 μg/ml sericin groups contained aggregated particles, while the 50 and 100 μg/ml sericin groups produced dispersed particles with more uniform particle size. This result indicates that the concentration of sericin affected the HAP mineralization process. According to previous reports, the nucleation of HAP is triggered by the anionic side chains in the protein combining with calcium ions (Kundu et al., 2008; Wang et al., 2010; Chen et al., 2016). Therefore, as the sericin content increased 10–100 μg/ml, the amount of acidic amino acids also increased, promoting the nucleation of HAP.

The effect of mineralization time on HAP nucleation was explored (Figure 1B). When the mineralization time was increased to 8 h, the mineralized HAP crystals in the 100 μg/ml sericin group showed a nanoplate-like structure. The nanoplate crystals were approximately 50–70 nm in length and 15–20 nm in width, which is close to the morphology of natural HAP (Gao et al., 2016). The high-resolution image revealed d-spacings of 0.334 and 0.272 nm, corresponding to the distance of the (002) plane and (211) plane of HAP, respectively (Zheng et al., 2019). EDS analysis showed that C, N, O, Ca, and P were evenly distributed on Ser100-HAP (Figure 1C). The molar ratio of Ca to P was 1.60, which is close to the 1.67 value of natural HAP (Xia et al., 2021).

XPS analysis was carried out to detect the surface atomic composition and chemical characteristics of the Ser-HAP samples. The XPS spectrum of Ser-HAP shows three strong peaks at 290, 400, and 530 eV, which are attributed to C 1s, N 1s, and O 1s, respectively (Figure 1D). In addition, the Ser-HAP sample exhibited photoelectron peaks at 134 eV (P 2p), 190 eV (P 2s), and 347 eV (Ca 2p), which correspond to the characteristic peaks of crystalline HAP. The carbon peaks of the C-C bond (C 1s, 284.8 eV) and the nitrogen peak of C−NH_2_ (N 1s, 400 eV) were also observed for the Ser10-HAP, Ser50-HAP, and Ser100-HAP groups, indicating that sericin was integrated into HAP (Teotia et al., 2017).

The chemical structure of the Ser-HAP samples was characterized by FTIR spectroscopy (Figure 1E). The absorption peaks located at 1136, 1064, and 985 cm^−1^ are attributed to the P-O stretching vibration mode and are the characteristic absorption peaks of HAP (Cai et al., 2010). Moreover, as the sericin content increased from 10 μg/ml (Figure 1E, Ser0-HAP) to 100 μg/ml (Figure 1E, Ser100-HAP), the amide I peak shifted from 1640–1651 cm^−1^, indicating that more sericin was integrated into the HAP particles (Radha et al., 2018; Veiga et al., 2020; Yu et al., 2021).

XRD analysis was used to determine the phase composition of the Ser-HAP sample. A small peak around 11.66° was observed, which may be due to the HAP lattice as previously reported (Radha et al., 2018). The sharp peaks at 21.2° and 32.1° are assigned to the (002) and (211) planes of HAP (Figure 1F). The intensity of these two peaks increased with the sericin concentration, indicating that sericin affected the nucleation of HAP. All the results showed that the sericin content plays an essential role in the nucleation of HAP particles, and sericin was successfully introduced into HAP (Takeuchi et al., 2005; Cai et al., 2010; Liu et al., 2013; Yang et al., 2014; Zhang et al., 2019; Veiga et al., 2020).

### Culture and Phenotypic Characterization of hPDLSCs

**FIGURE 2:**
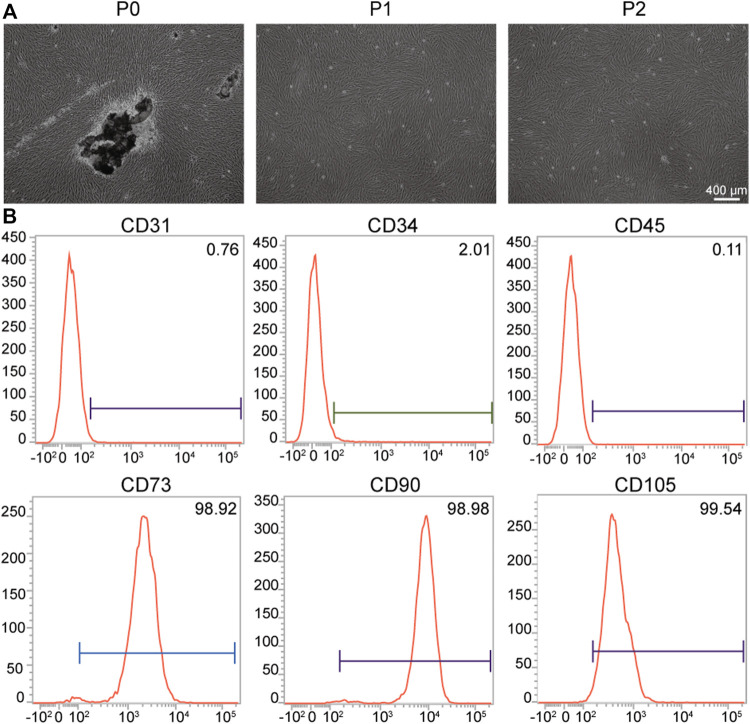
Culture and identification of hPDLSCs. **(A)** Morphology of hPDLSCs; **(B)** Flow cytometry analysis of the indicated cell surface marker expression in hPDLSCs.

hPDLSCs, a type of mesenchymal stem cells (MSCs) derived from the periodontal tissue, exhibit strong differentiation into osteoblasts and have therefore attracted increasing attention in the field of periodontal bone tissue regeneration (Zhao et al., 2019). As shown in Figure 2A, the black part was human periodontal ligament tissue, and the cells that emerge from the human periodontal tissue present a spindle shape. The results of flow cytometry show that the MSC surface markers CD73 (98.92%), CD90 (98.98%), and CD105 (99.54%) were positive. However, low expressions of the hematopoietic stem cell surface markers CD31 (0.76%), CD34 (2.01%), and CD45 (0.11%) were observed (Figure 2B). All of the results suggest that the isolated hPDLSCs had the potential for osteogenic differentiation and were not mixed with hematopoietic stem cells or endothelial cells (Liu et al., 2020; Peng et al., 2021; Rao et al., 2021).

### Cytocompatibility of Sericin and Ser-HAP Particles

**FIGURE 3:**
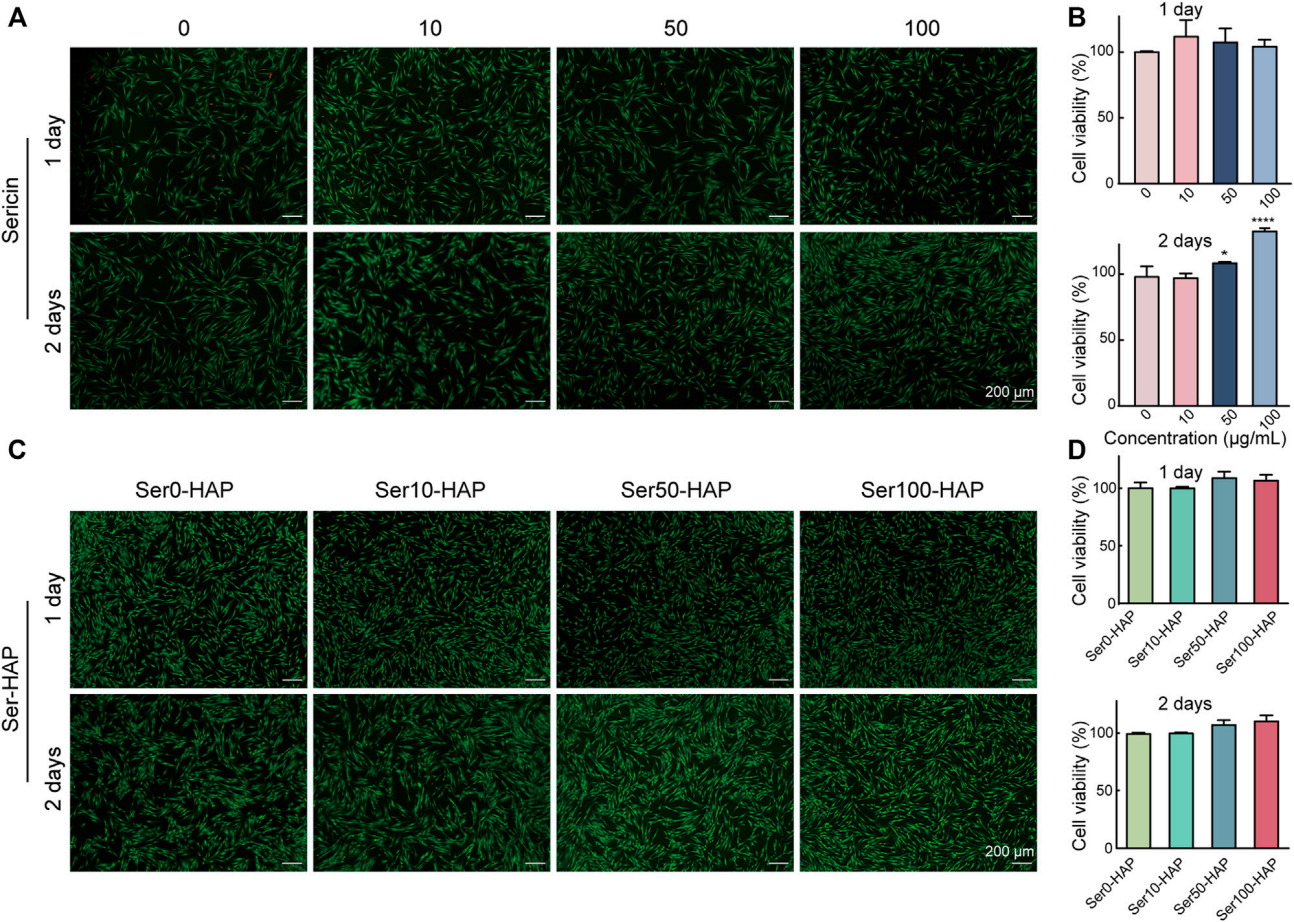
Cytocompatibility of sericin solution and the Ser-HAP particles. **(A)** Live/Dead staining and **(B)** CCK-8 assay of sericin at 1 and 2 days; **(C)** Live/Dead staining and **(D)** CCK-8 assay of Ser-HAP particles at 1 and 2 days.

To evaluate the cell biocompatibility of sericin and Ser-HAP particles in-depth, Live/Dead staining and a CCK-8 assay were used to examine the effects of sericin and Ser-HAP particles on the viability of hPDLSCs. Following incubation for 24 h, there was no significant difference in the viability of hPDLSCs treated with different concentrations of sericin (10, 50, and 100 μg/ml) compared with the control group. After incubation for 48 h, the cell viability of the 50 μg/ml group was slightly higher (*p < 0.05) than those of the 0 and 10 μg/ml groups, and the cell viability of the 100 μg/ml group was the highest (**p < 0.01) (Figures 3A,B). We speculate that in a serum-free environment, the amino acids in sericin serve as an energy source for cell metabolism and promote the growth of hPDLSCs (Lamboni et al., 2015; Wu et al., 2021). As shown in Figures 3C,D, Live/Dead staining and the CCK-8 assay indicated that Ser-HAP particles did not significantly affect the cell viability of hPDLSCs. Sericin and Ser-HAP were found to have excellent cytocompatibility, which lays the foundations for the application of sericin-based scaffolds in periodontal tissue engineering (He et al., 2017).

### Effects of Ser-HAP Particles on the Osteogenic Differentiation of hPDLSCs

**FIGURE 4:**
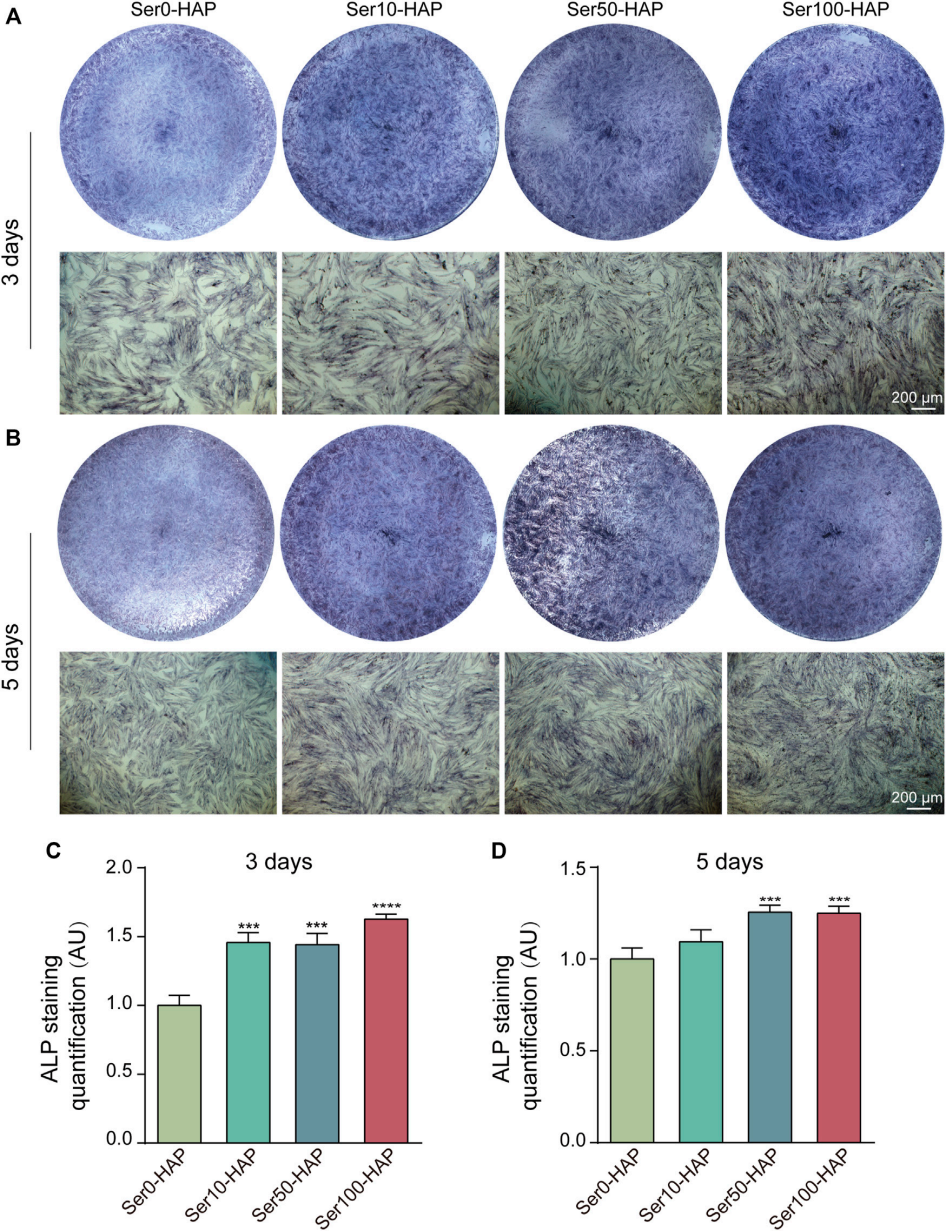
ALP staining of hPDLSCs after culture with Ser-HAP for 3 days **(A)** and 5 days **(B)**; Quantitative analysis of ALP staining at 3 **(C)** and 5 days **(D)**.

HAP, the main artificial substitute for alveolar bone, has the ability to promote the differentiation of stem cells into osteoblasts (Pan et al., 2020). ALP staining was used to detect the osteoinductive effect of different Ser-HAP samples on hPDLSCs. As shown in Figures 4A,B, the ALP secreted by hPDLSCs was stained blue. The results show that the expression of ALP in the Ser0-HAP, Ser10-HAP, Ser50-HAP, and Ser100-HAP groups gradually increased on days 3 and 5 (Figures 4A–D). All the results indicate that the osteogenic properties of Ser-HAP were improved in comparison to pure HAP (Ser0-HAP group). This observation is attributed to sericin protein promoting the formation of nanoscale HAP particles, which significantly increased the surface area to volume ratio of HAP and thus accelerated the release rates of calcium and phosphorus.

### Surface Topography of PVA/Ser-HAP Membranes

**FIGURE 5:**
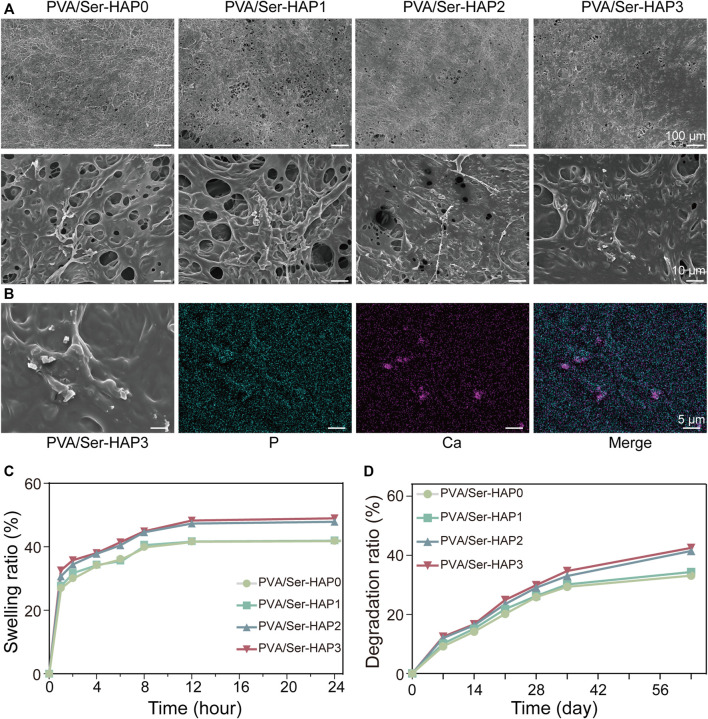
Surface morphology and characterization of PVA/Ser-HAP membranes. **(A)** SEM micrographs of PVA/Ser-HAP membranes; **(B)** Elemental mapping of P and Ca in the PVA/Ser-HAP3 membrane; **(C)** Swelling and **(D)** degradation of the PVA/Ser-HAP membranes.

Based on the available data, Ser100-HAP particles were selected for preparing PVA/Ser-HAP membranes by mixing with 4% (w/v) polyvinyl alcohol and 1% (w/v) sericin. FESEM images were acquired to reveal the morphology of the PVA/Ser-HAP membranes. Figure 5A shows that both the pure PVA/Ser membrane and the PVA/Ser-HAP membrane had rough surfaces, indicating that the introduction of Ser-HAP did not significantly affect the surface morphology of the membrane. Previous studies have shown that rough surfaces are conducive to improving the adhesion and migration of hPDLSCs on composite membranes during periodontal bone repair (Deligianni et al., 2001). In addition, EDS mapping showed that Ca and P were homogenously distributed in the PVA/Ser-HAP3 membrane (Figure 5B). And it has been reported that calcium-enriched areas may be the active site of mineralization during bone formation (Huang et al., 2020).

### *In Vitro* Hydrophilicity and Degradation of PVA/Ser-HAP Membranes

The ideal GTR membrane should retain an appropriate amount of water, which allows it to mimic the physiological functions of the original tissue and promote the proliferation and adhesion of cells (Prakash et al., 2019). The swelling kinetics of the PVA/Ser-HAP membranes were therefore explored (Figure 5C). All of the membranes swelled rapidly in the few minutes before reaching equilibrium. There was no significant difference in the swelling kinetics of the PVA/Ser-HAP0 and PVA/Ser-HAP1 membranes. During the soaking process of PVA/Ser-HAP membranes, with the release of Ser100-HAP NPs, the pores of the polymer PVA/Ser-HAP membranes were unblocked. Therefore, the swelling performance of PVA/Ser-HAP2 and PVA/Ser-HAP3 changed slightly with increasing HAP content (Prakash et al., 2019). The complete degradability of the membrane is very important for clinical applications (Liu et al., 2020). The degradation behavior of the PVA/Ser-HAP membranes was measured by weight loss (Figure 5D). All membranes showed similar degradation rates during the first 3 weeks, then the weight losses of the membranes reached 33%–42% at 9 weeks. The PVA/Ser-HAP3 membrane shows slightly higher degradation due to the release of more HAP. These results indicate that the PVA/Ser-HAP membranes were appropriately hygroscopic and degradable, which are important properties for their application in periodontal tissue engineering (Catori et al., 2021).

### Cytocompatibility of PVA/Ser-HAP Membranes

**FIGURE 6:**
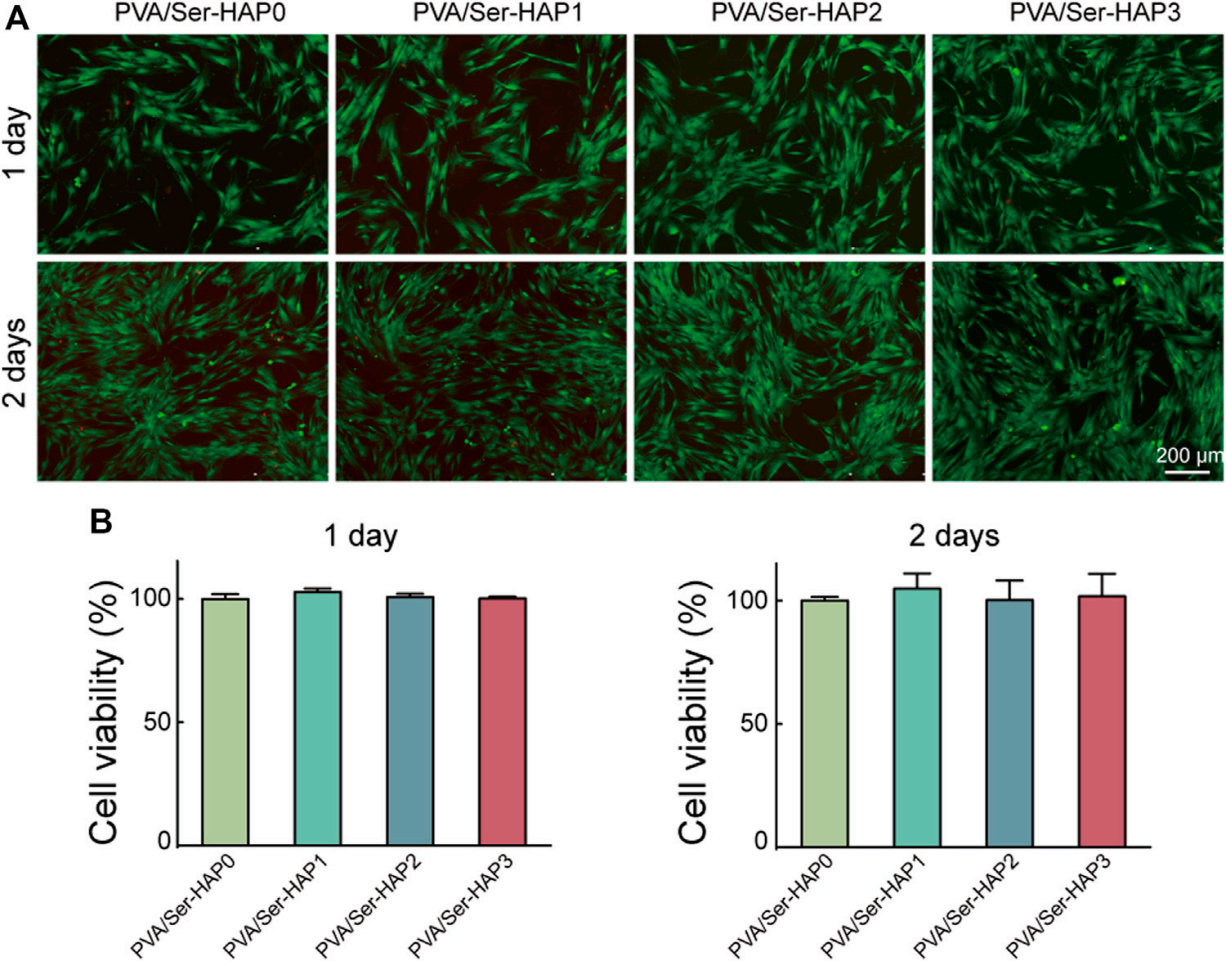
Biological compatibility of PVA/Ser-HAP membranes. Live/Dead staining **(A)** and CCK-8 assay **(B)** of hPDLSCs on the PVA/Ser-HAP membranes.

Biological compatibility is crucial for the application of GTR membranes in periodontal tissue regeneration engineering. The viability of hPDLSCs seeded on PVA/Ser-HAP membranes for 1 or 2 days was investigated using Live/Dead staining. Figure 6A shows the morphology of hPDLSCs adhered to various membranes, and indicates good cell vitality. Following the culture of hPDLSCs on the four PVA/Ser-HAP membranes for 2 days, the number of cells increased significantly, indicating that they have excellent biocompatibility (Nitti et al., 2021). In addition, CCK-8 analysis showed no significant difference in the cell viability after culture on the four PVA/Ser-HAP membranes for 1 or 2 days (Figure 6B).

### Cell Morphology of hPDLSCs on PVA/Ser-HAP Membranes

**FIGURE 7:**
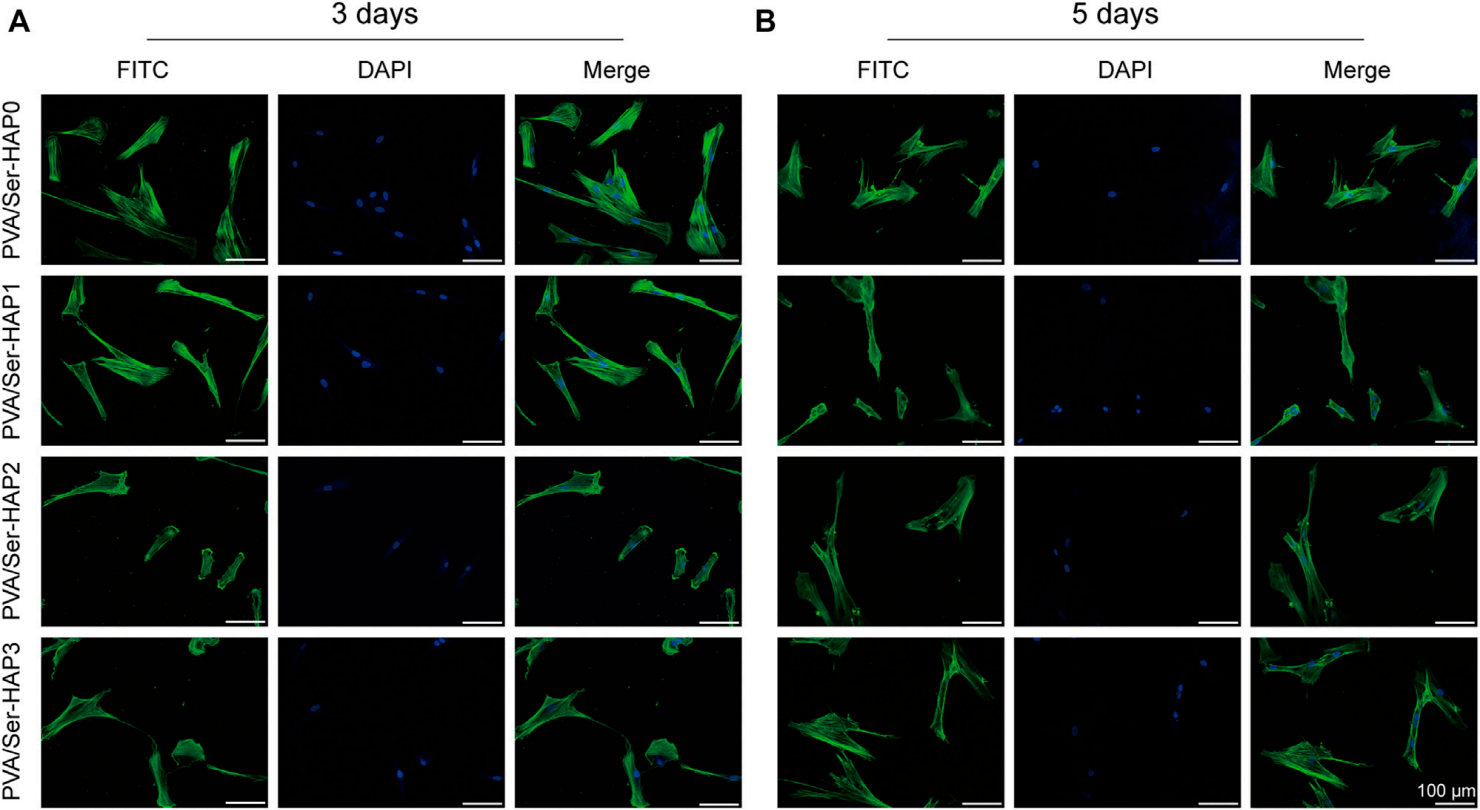
Morphology of hPDLSCs after seeding on PVA/Ser-HAP membranes for 3 days **(A)** and 5 days **(B)**. Green, cytoskeleton stained with phalloidin; Blue, nuclei stained with DAPI.

After 3 or 5 days of culture, the cells were stained for F-actin and nuclei, and then the morphology of the hPDLSCs on the PVA/Ser-HAP membranes was characterized by confocal microscopy (Wu et al., 2021). Figure 7 shows that the hPDLSCs on the membranes were spindle-shaped, and there was no significant difference in cell morphology between the different groups. Generally, spindle-shaped hPDLSCs with extended pseudopods indicate strong membrane attachment, while round hPDLSCs without pseudopods indicate almost no affinity for the membrane (Huang et al., 2020). These results indicate that the PVA/Ser-HAP membrane has good biocompatibility, and the cells adhered to the membrane surfaces well. These characteristics demonstrate the potential for application of the membranes in periodontal regeneration.

### Osteogenic Differentiation of hPDLSCs Cultured With PVA/Ser-HAP Membranes

**FIGURE 8:**
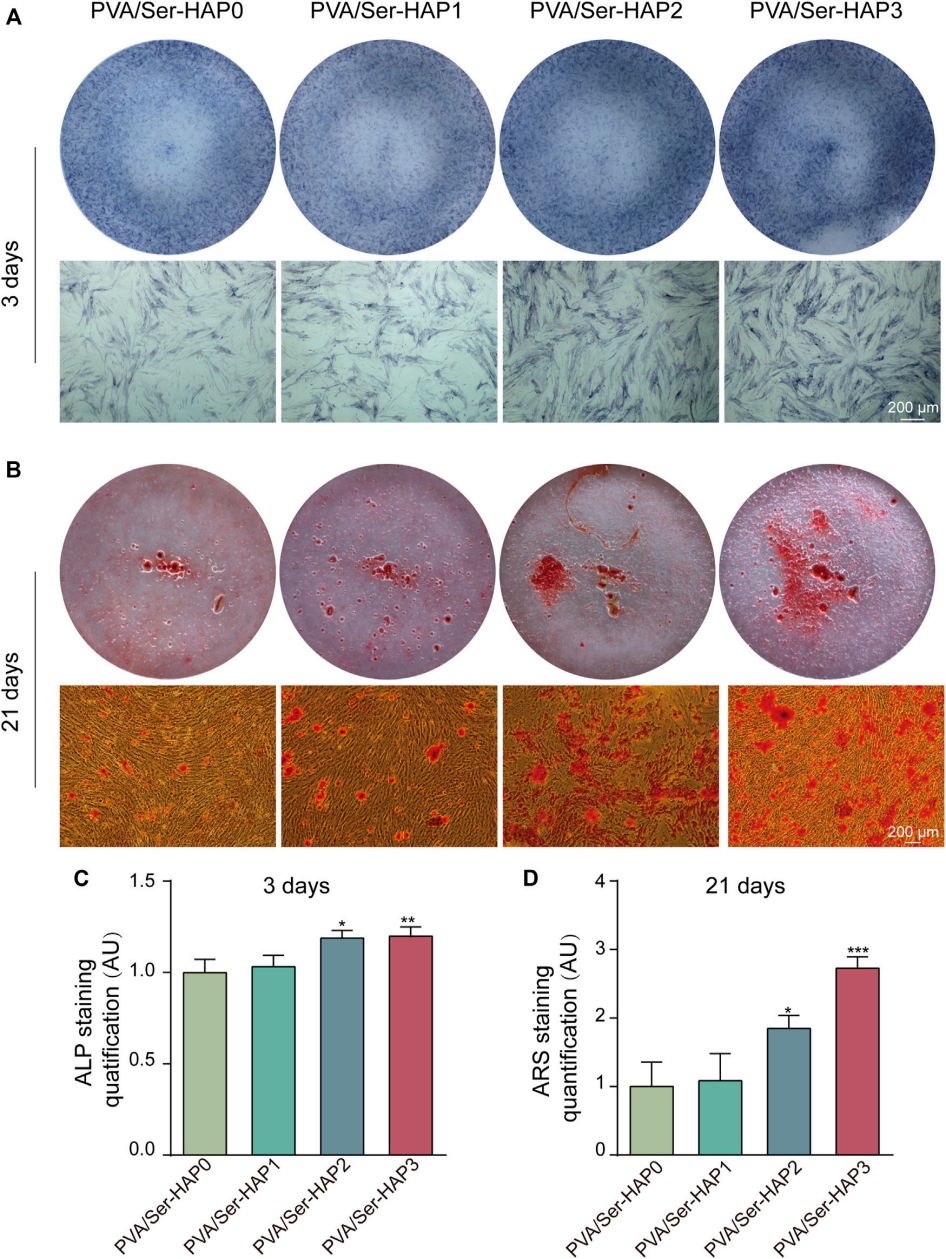
**(A)** ALP staining of the hPDLSCs following cultured by extracts of PVA/Ser-HAP membranes for 3 days; **(B)** Alizarin red staining of the hPDLSCs after cultured by extracts of the membranes for 21 days; **(C)** Quantitative analysis of ALP staining at 3 days; **(D)** Quantitative analysis of Alizarin red staining at 21 days.

ALP staining, ARS, and RT-qPCR were used to analyze the osteogenic differentiation of hPDLSCs seeded on the four membranes. Following 3 days of treatment, the expression of ALP in the PVA/Ser-HAP2 and PVA/Ser-HAP3 groups was significantly up-regulate compared with that of the PVA/Ser-HAP0 group (Figures 8A,C). These findings indicate that the PVA/Ser-HAP membranes markedly promoted the early osteogenic differentiation of hPDLSCs. As a late marker of the mineralization stage, ARS staining is used to study the production of calcium nodules (Zhu et al., 2020). As shown in Figures 8B,D, after co-cultured with hPDLSCs for 21 days, the PVA/Ser-HAP3 group showed the most apparent calcium mineral nodules, while the PVA/Ser-HAP0 group showed fewer calcium mineral nodules. These findings indicate that PVA/Ser-HAP membranes effectively promote the late osteogenic differentiation of hPDLSCs.

**FIGURE 9:**
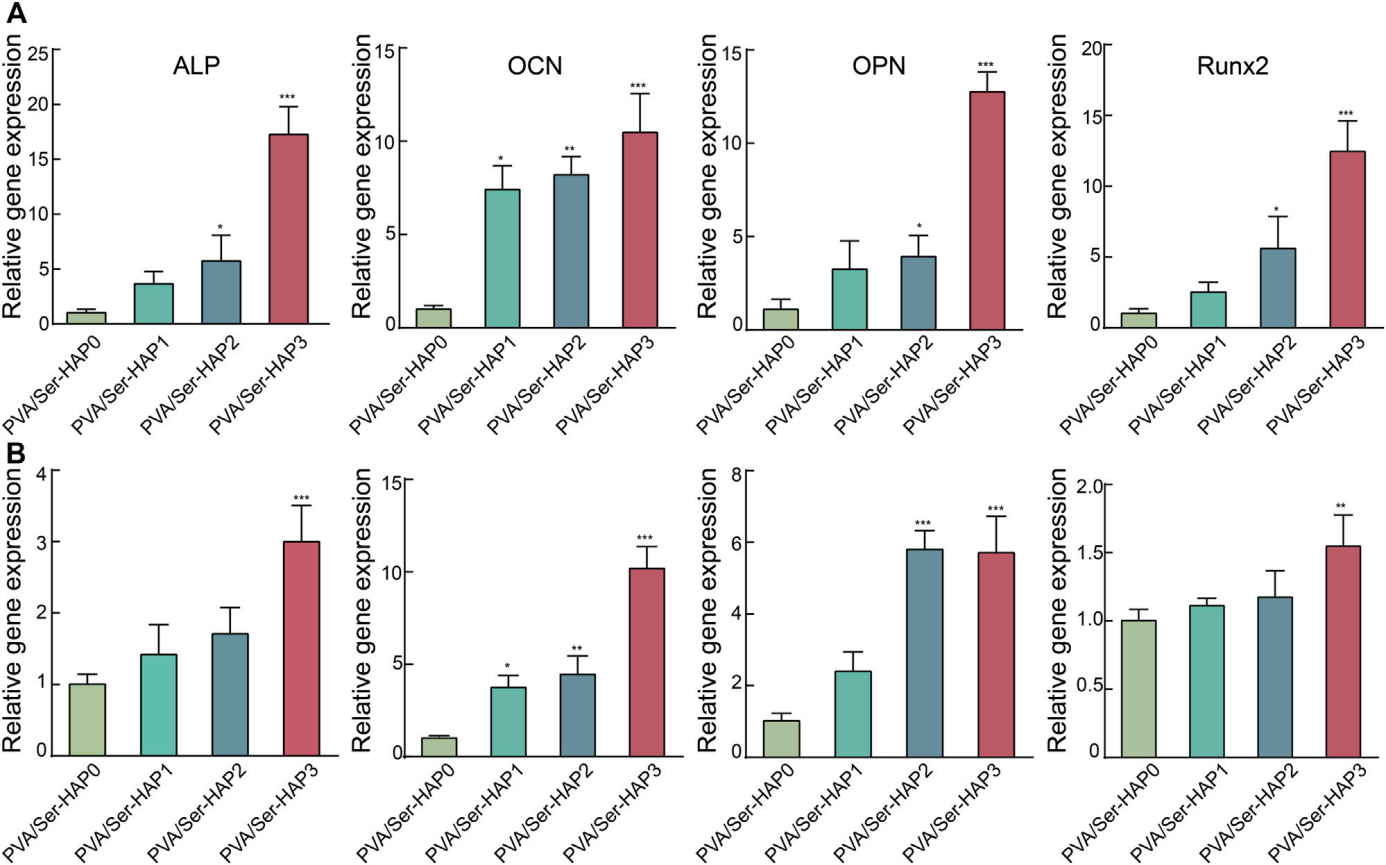
RT-qPCR analysis of the ALP, OCN, OPN, and Runx2 expression of hPDLSCs following cultured by extracts of the PVA/Ser-HAP membranes for 3 days **(A)** and 5 days **(B)**.

HAP has outstanding osteoconductive properties (Deligianni et al., 2001; Maleki-Ghaleh et al., 2021). A previous study reported that HAP promoted osteogenic differentiation of BMSCs through upregulation of ALP, OPN, and OCN (Yang et al., 2014). In addition, Runx2, a vital transcription factor for osteogenic differentiation, is closely related to the p38 MAPK pathway and can be triggered by HAP during osteoblast differentiation (Hiragami et al., 2005). To further verify the osteoinductivity of the PVA/Ser-HAP membranes, the expression of osteogenic genes by hPDLSCs cultured on the membranes was determined. Figure 9 shows that the expression of osteogenic genes, including Runx2, ALP, OPN, and OCN, were significantly upregulated on the PVA/Ser-HAP3 membrane at 3 and 5 days, compared with on the PVA/Ser-HAP0 membrane (*p* < 0.01). The results indicate that the PVA/Ser-HAP membrane induces the expression of osteogenic proteins and genes and has the potential to induce the repair of alveolar bone defects.

## Conclusion

We successfully synthesized Ser-HAP nanoparticles using sericin as an organic template. The sericin concentration and mineralization time were found to affect the nucleation of HAP. Ser-HAP nanoparticles exhibited excellent biocompatibility and promoted osteogenic differentiation of hPDLSCs. The Ser100-HAP nanoparticles were subsequently combined with PVA, and biomimetic PVA/Ser-HAP membranes were prepared by repeated freezing and thawing. Further studies showed that PVA/Ser-HAP membranes did not affect the viability of hPDLSCs. Most importantly, ALP staining, ARS, and RT-qPCR detection showed that PVA/Ser-HAP membranes promoted osteogenic differentiation of hPDLSCs. These findings suggest that PVA/Ser-HAP membranes have excellent potential for application in periodontal regeneration and repair therapy.

## Data Availability

The original contributions presented in the study are included in the article/Supplementary Material, further inquiries can be directed to the corresponding authors.
